# Analysis of Particles’ Size and Degree of Distribution of a Wooden Filler in Wood–Polymer Composites

**DOI:** 10.3390/ma14216251

**Published:** 2021-10-20

**Authors:** Iwona Michalska-Pożoga, Marcin Szczepanek

**Affiliations:** 1 Faculty of Mechanical Engineering, Koszalin University of Technology, Raclawicka 15-17, 75-620 Koszalin, Poland; 2 Faculty of Marine Engineering, Maritime University of Szczecin, Wały Chrobrego 1-2, 70-500 Szczecin, Poland; m.szczepanek@am.szczecin.pl

**Keywords:** image analysis, particles’ size, degree of filler’s distribution, wood–polymer composites (WPCs)

## Abstract

In wood–polymer composites (WPCs), regardless of the origin of the filler and its dimensions, their significant role in changing the properties of the WPCs’ material was found. Given the above, it is of particular importance to determine the size of the wood filler particles after their production. In addition, it is also important to determine the degree of distribution of the filler in the polymer matrix. The methodology for determining particle size and distribution is complex, even when using image analysis computer systems. This article presents the application and implementation of the multi-stage procedure for determining the size of wood particles and the degree of their distribution in the WPCs by means of image analysis using a numerical calculation program. The procedure, co-authored by the researchers at the Koszalin University of Technology and School of Mechanical and Materials Engineering, is published in the Industrial Crops and Products 2016 Comparing the results obtained for the PP/Lignocel 3-4 and PP/Lignocel C120 composites produced under highly different conditions in the target zone, it was found that the degree of the component distribution in the polymer matrix was significantly influenced by the width of the target gap. In both cases, the best homogeneity of the material and a good distribution of the filler in the polymer matrix was achieved within the parameters that have a mild effect on the material and allow it to stay longer in the plasticizing system, i.e., Ws = 1.0–3.0 mm with simultaneous impact medium to high speed in the range n = 26–40 rpm.

## 1. Introduction

The production of wood–polymer composites (WPCs) is currently at the level of over 1.5 million tonnes worldwide (according to the German Nova-Institute). Polymer composites with at least one biological material component have attracted the attention of researchers worldwide. It was found that the properties of the obtained polymer composites depend not only on their composition but also on the size and form of individual components used for their preparation [1,2,3,4,5,6,7]. This finding is important not only in the case of materials reinforced with fillers but also in the case of single-material composites referred to as monopolymer composites (MPCs) or single-polymer composites (SPCs) where the material exists in two different forms [8]. Wood–polymer composites (WPCs) are one material for which the size, shape, and amount of the dispersed phase are particularly important. The wood fraction in the WPCs can consist of particles of different forms, ranging from dusty wood flour, through fine sawdust, to shavings and scraps of a few millimeters [9,10]. In addition, the wood fraction may consist of hardwood, softwood, or mixed pieces of different origins (softwood or hardwood) in the case of recycled wood. Coniferous wood is marked by characteristics other than hardwood, as the softwood fraction is more susceptible to crushing during processing than hardwood.

As a result of their interaction with the elements of the plasticizing system, the size of the particles changes causing, in turn, the changes in the properties of the obtained composite [11,12]. Hence, determining the dimensions of the wood particles in both the raw material and the finished product is a significant issue. For the raw material, the measurement is simple and requires preparing a chip sample, spreading it in a single layer, taking a picture, and analyzing the image. However, the procedure is not as simple in the case of the final product. Unfortunately, wood particles (dispersed phase) visible in the images often accumulate to form agglomerates, which can seriously affect the results of the image analysis. Another serious problem is the overlapping of wood fragments in the extruded mass [13], which creates a challenge for researchers and the computerized image analysis system. This problem can be controlled when selecting the pieces of wood and determining the size during image analysis. However, this process requires experience and knowledge. Correct results can be obtained by paying close attention to the outlines of individual wood particles, but it may significantly extend the image analysis procedure.

Studies in the literature indicate that the systems that automatically analyze the image have implemented procedures favoring large (usually) or small particles [14,15,16]. Therefore, the obtained results do not correspond to the actual state. The article presents an image analysis of the wood–polymer composite samples using a multi-stage image analysis methodology to determine the size of the wood fraction particles and the degree of their distribution in the WPCs.

## 2. Materials and Methods

### 2.1. Research Material

**Figure 1:**
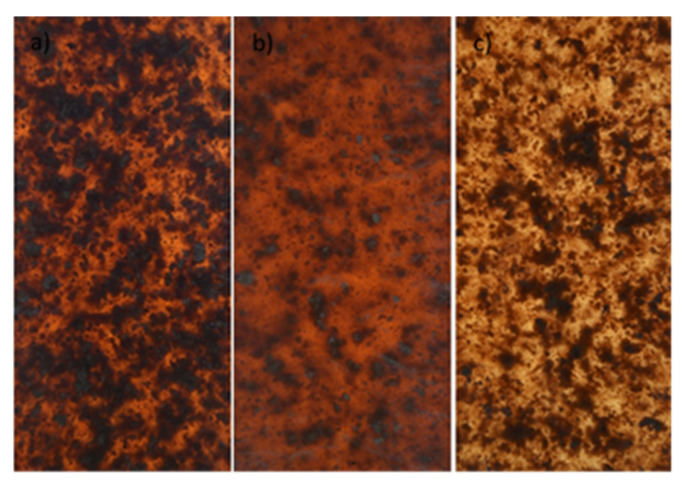
Examples of composite tiles with wood flour Lignocel C120 (PP/Lignocel C120).

**Figure 2:**
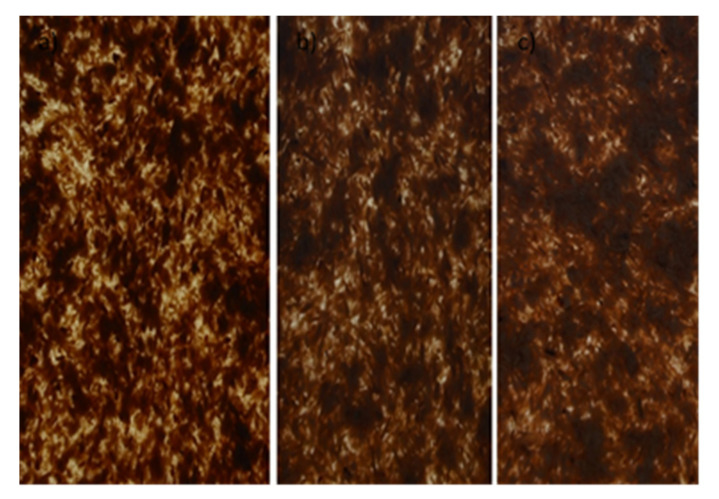
Examples of composite tiles with wood chips Lignocel 3-4 (PP/Lignocel 3-4).

The research material consisted of composite tiles (Figure 1 and Figure 2) comprised of a polymer matrix made of PP polypropylene and wood reinforcement in the form of wood flour and wood shavings. The material used for the composite matrix was polypropylene (PP) with the trade name Moplen HP456J produced by Basell Orlen Poliolefins Ltd. Płock, Poland., whose properties are summarized in Table 1.

**Figure 3:**
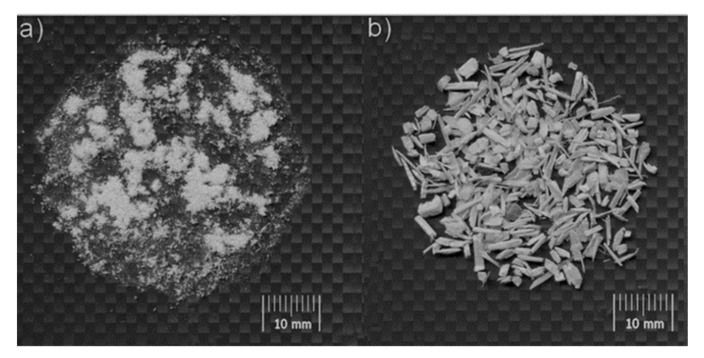
A real image of a coniferous wood filler: (**a**) wood flour Lignocel C120; (**b**) wood chips Lignocel 3-4.

**Figure 4:**
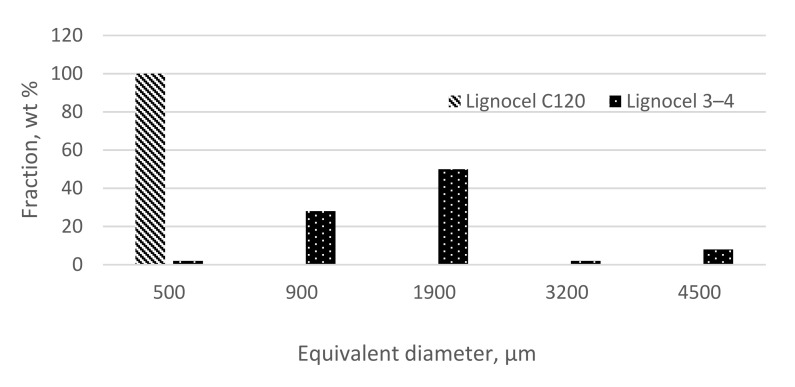
Graph showing the size distribution of the respondents’ wood chips: Lignocel C120 and Lignocel 3-4.

Coniferous wood with different granulation: wood flour with the trade name Lignocel C 120 (Figure 3a) with dimensions of 70–150 μm and chips with the trade name Lignocel 3-4 (Figure 3b) with dimensions of 1500–4500 μm produced by Rettenmaier und Söhne GmbH + Co. KG from Germany with the grain size distribution of the wood fraction shown in Figure 3 was used as reinforcement material. Figure 4 shows a graph showing the size distribution of the respondents’ wood chips.

Before mixing and extrusion, the wood material was dried at the temperature of 105 °C ± 5 °C for 4 h. The surface of the wood material was not modified.

### 2.2. Obtaining Composite WPCs

Composite plates were obtained in the extrusion process using a worm-disk plasticizing system with the technology presented in previous publications [18,19]. The temperature of the hot zone was a constant parameter t = 160 °C. At the same time, the rotational speed of the screw (and the disc) n and the width of the disc chink W_s_ were the variable factors (Table 2).

The screw-disc extruder is a prototype construction, developed and made at the Faculty of Mechanical Engineering of the Koszalin University of Technology, described by I. Michalska-Pożoga and T. Rydzkowski [20,21,22,23,24].

### 2.3. Obtaining Composite WPCs

**Figure 5:**
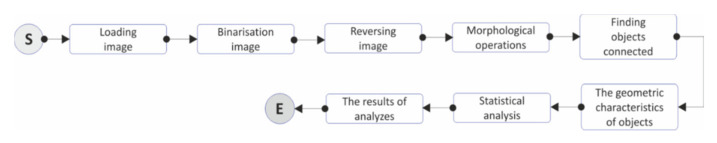
Elaborated algorithm of an evaluation of graphic images (pictures) of WPCs [1].

To determine the particle size and the degree of homogeneity of the distribution of the wood fraction in the form of flour and chips in the polymer matrix, image analysis was carried out using the MATLAB package, version 8.3.0.532. Elaborated methodology of image analysis of the WPCs containing filler in the form of flour and wood shavings is composed of eight steps (Figure 5).

Photographic documentation of composite tiles with dimensions of 130 mm × 60 mm (sample area 7800 mm^2^) was made. Images of composite tiles were recorded with a NIKON 5100 digital camera with additional, multi-point LED lighting. Images with a resolution of 300 dpi were obtained. The image processing algorithm uses a two-dimensional analysis of the registered images [1]. The methodology presented by the author of the article in the publication from 2016 [1] was used to analyze the images of composite tiles.

This article presents the results of the analysis of the distribution of Lignocel C120 wood flour and Lignocel 3-4 wood shavings in a polypropylene matrix (marking respectively: PP/Lignocel C120; PP/Lignocel 3-4). The analysis was carried out for the parameters of the extrusion process with disc zone chink (W_s_) and rotational speed (n) selected in such a way as to cause extreme phenomena in the disc zone of the screw-disk extruder. The focus was on the parameters causing the occurrence of high and low shear rates, respectively:

- Simultaneous influence of small disc chink widths and medium rotational speeds, i.e., W_s_ = 0.3 mm and n = 26 rpm (γ ^·^ = 800 s^−1^), under conditions of intense short-term shear-mixing interaction.
- Simultaneous influence of medium disc chink widths and medium rotational speeds, i.e., W_s_ = 1.7 mm and n = 26 rpm (γ ^·^ = 400 s^−1^), under conditions of intense short-term shear-mixing interaction.
- Simultaneous influence of large width of the disc chink and medium rotational speeds, i.e., W_s_ = 3.0 mm and n = 26 rpm (γ ^·^ = 70 s^−1^), under mild long-term mixing conditions.

Moreover, the influence of rotational speed change n (rpm) = 12, 26, 40 and W_S_ = 1.7 mm, on the homogenization of the WPCs and the particle size distribution of the wood fraction was analyzed. The analysis was performed on 18 samples.

The presented article is a practical application of the method and the model described in the 2016 article. The procedure, co-authored by research workers of the Koszalin University of Technology and the College of Mechanical and Material Engineering, was published in Industrial Crops and Products 2016 [1].

## 3. Results

The WPCs manufactured according to the methodology included in chapter 2 of this article were analyzed. In the first stage, the analysis of the WPCs (PP/Lignocel C120 and PP/Lignocel 3-4) produced at different values of shear rate was carried out.

**Figure 6:**
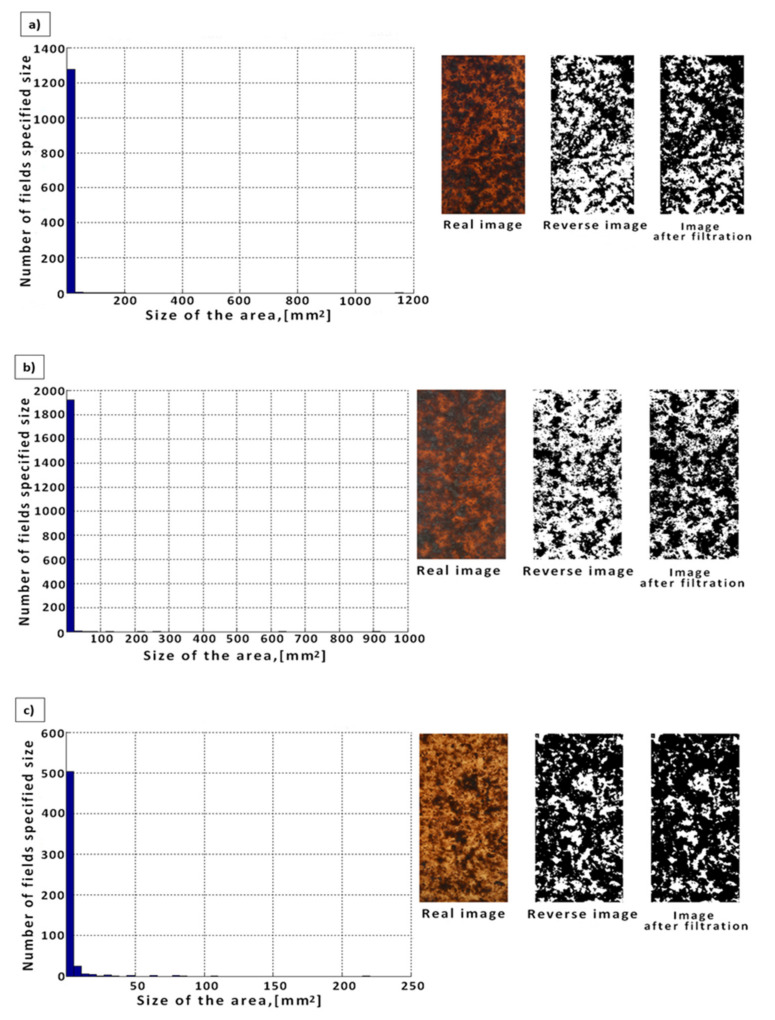
Histogram of the determined numbers and areas of agglomerates for WPCs with the addition of wood flour (PP/Lignocel C120) obtained under the conditions of (**a**) high shear rate values (γ ^·^ = 800 s^−1^), (**b**) medium shear rate values (γ ^·^ = 400 s^−1^), and (**c**) low shear rate values (γ ^·^ = 70 s^−1^).

Analyzing the WPCs with a fraction of wood flour (PP/Lignocel C120) obtained under conditions of short-term, intense shear-mixing interaction, where the shear rate was approximately 800 s^−1^, many clearly visible large clusters of wood flour accumulating in the form of agglomerates of various sizes were found (Figure 6a). In this case, 1300 agglomerates with an area of up to 40 mm^2^ were identified, 8 with an area between 40 and 75 mm^2^ and single ones up to 200 mm^2^, which in total represents approximately 33% (Table 3) of the sample area in relation to its total area. In the next step, when analyzing the samples subjected to the conditions causing the average values of the shear rate (400 s^−1^), some large clusters of wood flour were found (Figure 6b). In this case, aggregates with an area ranging from 0.0011 to 20 mm^2^ were identified, including 1200 aggregates with an area of up to 2.5 mm^2^, 25 with an area between 2.5 and 5 mm^2^, 8 with an area between 5 and 7.5 mm^2^, 4 with an area between 7.5 and 10 mm^2^, and the remaining singles above 10 mm^2^, which is in total about 10% (Table 3) of the sample area in relation to its total area. On the other hand, when analyzing the WPC obtained under the conditions of a mild, long-term mixing action, where the shear rate was approximately 70 s^−1^ on their surface, similar to the previously analyzed composites, many agglomerates were found, but with a smaller surface area (Figure 6c). Specifically, 520 agglomerates with an area of up to 7 mm^2^, 18 with an area between 7 and 14 mm^2^, 8 with an area between 14 and 22 mm^2^, and 4 with an area between 22 and 29 mm^2 w.^ The remaining individual agglomerates with an area of more than 30 mm^2^ were identified, which, in total, accounts for about 20% (Table 3) of the sample area.

**Figure 7:**
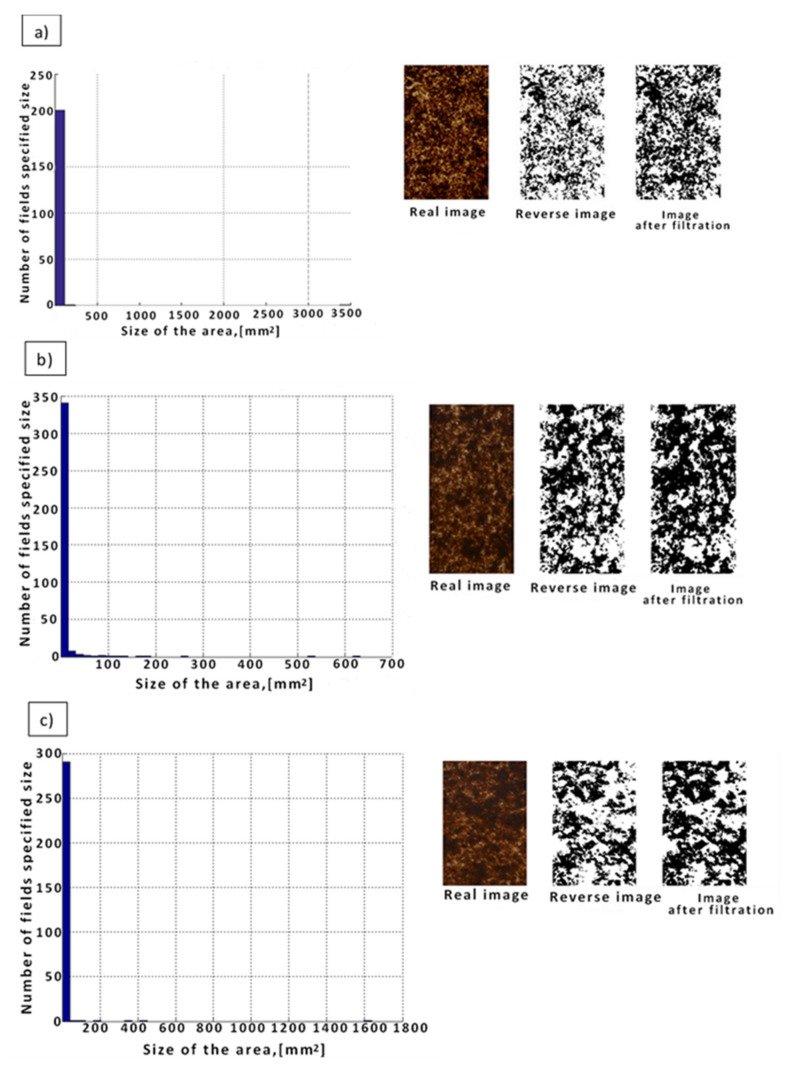
Histogram of the determined numbers and areas of agglomerates for WPCs with the addition of wood shavings (PP/Lignocel 3-4) obtained under the conditions of (**a**) high shear rate values (γ ^·^ = 800 s^−1^), (**b**) medium shear rate values (γ ^·^ = 400 s^−1^), and (**c**) low shear rate values (γ ^·^ = 70 s^−1^).

When analyzing the WPCs reinforced in the form of wood chips (PP/Lignocel 3-4) under conditions of short-term, intense shear-mixing interaction, many clearly visible large aggregates of the filler in the form of aggregates of various sizes were found on their surface (Figure 7a). In this case, aggregates with an area ranging from 0.0015 to 250 mm^2^ were identified, including 225 aggregates with an area of up to 60 mm^2^, 2 with an area between 60 and 135 mm^2^ and single ones above 135 mm^2^. On the other hand, when analyzing the WPC obtained under the conditions of a mild, long-term mixing effect, the presence of aggregates on their surface, as in the previous analyzed case, was found, but with a smaller surface area and more even distribution in the composite (Figure 7b). In this case, aggregates with an area ranging from 0.00025 to 200 mm^2^ were identified, including 289 aggregates with an area of up to 70 mm^2^, 2 with an area between 70 and 125 mm^2^, and the remaining individual agglomerates with an area of more than 125 mm^2^. The dependence of the sum of all aggregate areas in the tested composites and the percentage of the sum of aggregate areas to the total area of the tested sample on the shear rate are presented in Table 3 and Table 4.

Comparing the results obtained for the PP/Lignocel C120 composites produced under extremely different conditions in the target zone, it was found that the degree of the component distribution in the polymer matrix was significantly influenced by the width of the target gap. In the case of a composite with wood flour, in order to achieve the best possible distribution of the filler in the polymer matrix, it should be produced under mild, long-term mixing effects, i.e., with the simultaneous effect of large disc chink widths (in the range of W_s_ = 1.0–3.0 mm), and medium and high rotational speeds (in the range of n = 26–40 rpm). Under these conditions, rolling and circumferential distribution of the filler takes place. In the PP/Lignocel 3-4 composites, it has been found that the extent of distribution of the component in the polymer matrix is mainly influenced by the width of the disc chink, but not as pronounced as in the case of the wood flour filler. When comparing the composites obtained in significantly different processing conditions, i.e., low and high shear rate values, it was found that for processing composites filled with wood shavings, the best homogeneity of the material and good distribution of the filler in the polymer matrix is achieved within the parameters that mildly affect the material and allow a longer stay in the plasticizing system, i.e., large width of the disc chink in the range of W_s_ = 1.0–3.0 mm with the simultaneous influence of medium to high rotational speed in the range of n = 26–40 rpm. Using a large width of the disc gap and a low rotation speed also reduces the longitudinal dimensions of the long wood chips due to their breaking of the transverse dimensions resulting from defibration. This analysis confirms the exploratory research conducted by T. Rydzkowski [14].

In the case of WPCs, regardless of the form of filling, to achieve the best possible distribution of the filler in the polymer matrix, it should be produced under the conditions of gentle, long-lasting mixing action. The consequence of these activities will be obtaining satisfactory performance properties of the composite, as confirmed by the research published in Michalska-Pożoga [18].

**Figure 8:**
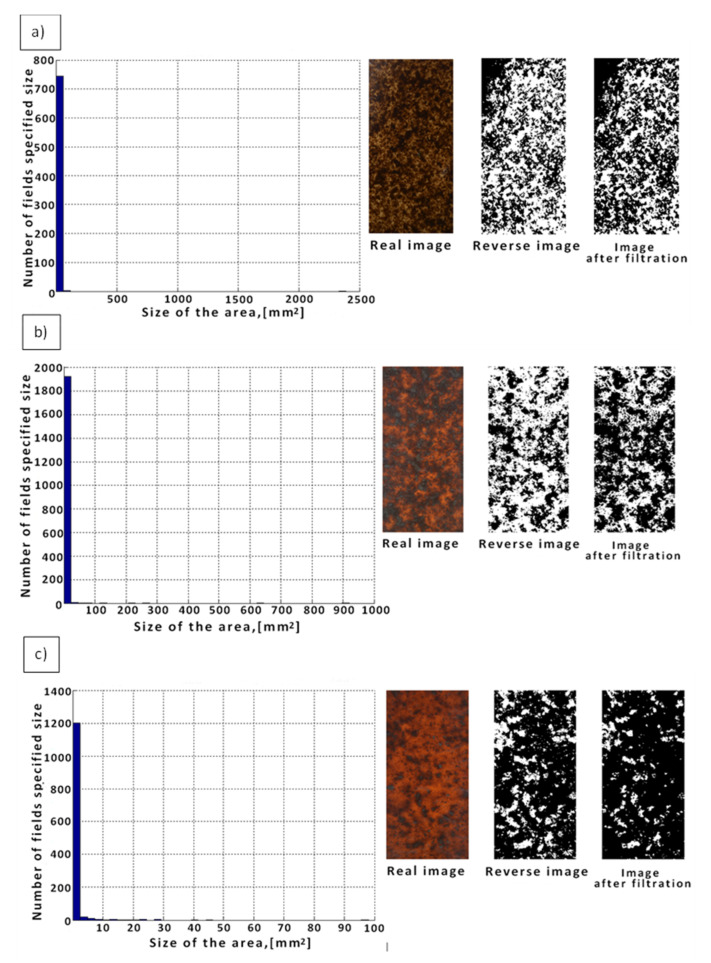
Histogram of the determined numbers and areas of agglomerates for WPCs with the addition of wood flour (PP/Lignocel C120) at constant: W_S_ = 1.7 mm, i = 35 w_t_%, and variable rotational speed: (**a**) n = 12 rpm, (**b**) n = 26 rpm, and (**c**) n = 40 rpm.

**Figure 9:**
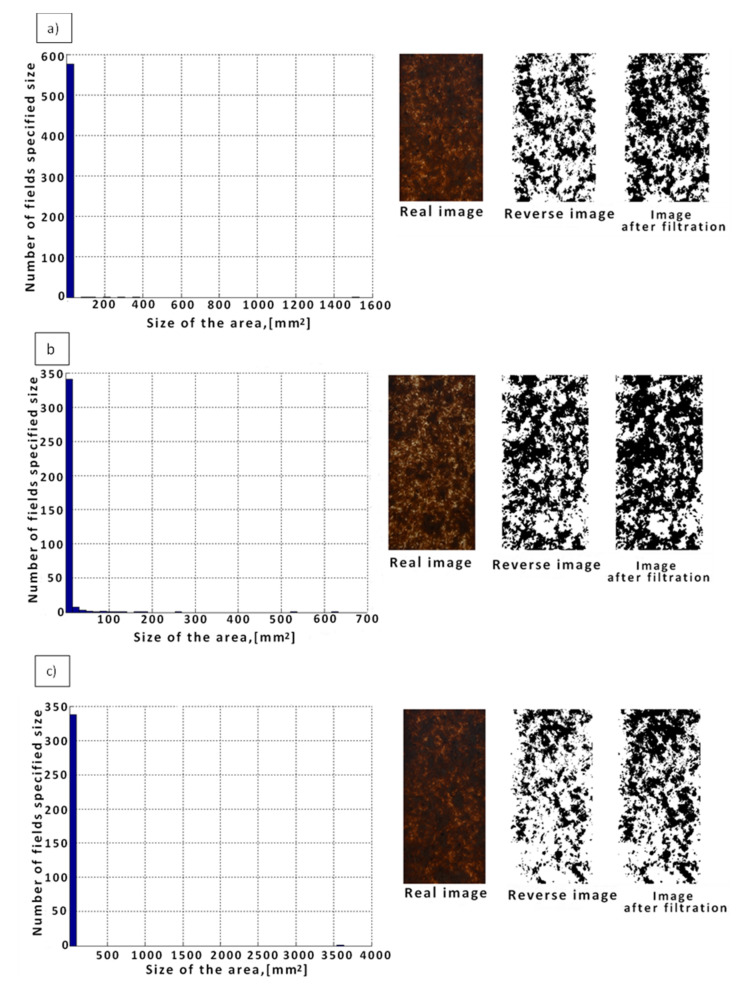
Histogram of the determined numbers and areas of agglomerates for WPCs with the addition of wood chips (PP/Lignocel 3-4) at constant: W_S_ = 1.7 mm, i = 35 w_t_%, and at variable rotational speed: (**a**) n = 12 rpm, (**b**) n = 26 rpm, and (**c**) n = 40 rpm.

In the next stage of the analysis, the impact of changing the rotational speed of the screw was determined, with the remaining parameters constant (the width of the disc chink (W_S_) and the amount of filler (i)) on the distribution of the filler in the form of wood flour and wood chips in the WPCs (Figure 8 and Figure 9).

When analyzing the WPCs (PP/Lignocel C120) obtained at n = 12 rpm, many clearly visible large agglomerates of the wood flour were found in the polypropylene matrix (Figure 8a). In this case, 750 agglomerates up to 50 mm^2^, 5 between 50 and 120 mm^2,^ and single agglomerates above 120 mm^2^ were identified. By increasing the value of the rotational speed to 26 rpm (Figure 8b), a decrease in the number of agglomerates and their surface was observed by an average of 20%. Another increase in the rotational speed from 26 to 40 rpm (Figure 8c) caused a decrease in the amount and size of agglomerates by about 80% in relation to the composites produced at the screw speed n = 12 rpm (Table 5).

When analyzing WPCs (PP/Lignocel 3-4) obtained at a rotational speed of 12 rpm, many clearly visible large aggregates of various sizes were found on their surface (Figure 9a). In this case, aggregates with a field area ranging from 0.0018 to 200 mm^2^ were identified, including 518 aggregates with an area of up to 40 mm^2^, 2 with an area between 75 and 150 mm^2,^ and single ones above 190 mm^2^. Percentage of it accounted for 48% of the total area. By increasing the value of the rotational speed to 26 rpm (Figure 9b), a decrease in the number of aggregates and their surface area was observed by an average of 30%. In this case, aggregates with an area ranging from 0.0025 to 100 mm^2^ were identified, including 340 agglomerates with an area up to 18 mm^2^, 8 with an area between 18 and 35 mm^2^, 3 with an area between 35 and 50 mm^2^, and others above 50 mm^2^. In the next step, increasing the rotational speed from 26 to 40 rpm (Figure 9c) caused an increase in the size of the aggregates with an amount similar to the amount for composites produced at the speed of 26 rpm. The size of the aggregates increased by an average of 80%. Aggregates with an area ranging from 0.0025 to 100 mm^2^ were identified, including 335 aggregates with an area of up to 90 mm^2^ and others above 90 mm^2^ (Table 5).

By analyzing the histograms presented in Figure 8 and Figure 9, it was found that the degree of distribution of the filler in the form of wood flour and wood shavings can be influenced by the adjustment of the rotational speed. By increasing its value, we produce material with a smaller number of agglomerates, and above all, with a smaller size. It may prove good mixing and distribution of the filler in the matrix. For the PP/Lignocel C120 composite at a rotational speed of 40 rpm, the size of the agglomerate is ten times the dimension (1.5 mm) of the maximum size of the filler (0.15 mm), while at the minimum speed, i.e., 12 rpm, the obtained the size of the agglomerate is forty-five times the dimension (0.07 mm) of the filler size. When the rotational speed is changed, the filler is distributed circumferentially. For the PP/Lignocel 3-4 composite, by increasing the rotational speed to its average values (up to 30 rpm), we obtain a material with a smaller number of aggregates, and above all, with a smaller surface area which proves the good distribution of the filler in the polymer matrix. At the minimum rotational speed of 12 rpm, the achieved linear dimension of the aggregate is approximately 1.5 times (6.3 mm) the length of a single filler particle (4.5 mm), at an average speed, i.e., approximately 26 rpm, the obtained average linear dimension of the aggregate equals the longitudinal dimension of a single chip, and at the maximum rotational speed, i.e., 40 rpm, the achieved linear dimension of the aggregate is approximately 2 times (9 mm) greater than the dimension of a single filler particle. In the case of wood chips, extrusion causes the grinding of the wood filler under the influence of shear stress [14,24,25].

## 4. Conclusions

1. By analyzing the degree of distribution of the fraction in the form of wood flour in a polypropylene matrix (PP/Lignocel C120), the presence of many aggregates of various sizes was found. The possibility of influencing the extrusion settings, that is, the width of the disc gap (Ws) and the rotational speed (n) on the size and the number of aggregates, was found. The simultaneous effect of high rotational speed and small width of the disc chink creates conditions for an intense shear-mixing effect, resulting in many large aggregates. On the other hand, the mild and long-term mixing effect created at a low rotational speed of the screw and a medium or large width of the disc gap allows for the production of a composite with a higher level of filler mixing in the polymer matrix (large numbers of small aggregates).
2. By analyzing the degree of distribution of the fraction in the form of wood chips in the polymer matrix (PP/Lignocel 3-4), the presence of many aggregates with a different surface area was found. The possibility of influencing the extruder settings, i.e., the width of the disc chink (Ws) and the rotational speed of the screw (n) on the size and number of aggregates, was found. The primary and significant influence in the case of filling in the form of wood chips is the mild effect of the target zone obtained with an average gap width and rotational speed on the processed material. Under such conditions, we obtain a material with a smaller number of aggregates, and above all, with a smaller surface area, which proves good mixing and distribution of the filler, as well as shortening the longitudinal dimensions of their particles, which facilitates the arrangement of chips in the flow direction.
3. The degree of distribution of the wood chip filler in the polymer matrix is influenced by the value of the disc chink, but not as intensely as in the case of the wood flour filler.
4. Based on the conducted analyzes, the applied method of analyzing images of the WPCs presented in the article is the optimal method for composites with low (i < 15% weight) and medium filled (15% weight < i < 50% weight) with a wood fraction in the form of chips and wood chips.

## Figures and Tables

**Table 1:** Characteristics of the material matrix (polypropylene PP Moplen HP456J) [17].

Parameters		Unit	Value
Melt flow rate (230 °C; 2.16 kg)		g/10 min	3.4
Density (w temp. 20 °C)		g/cm^3^	0.89–0.91
Tensile stress at break		MPa	23
Tensile strain at break		%	>500
Tensile stress yield point		MPa	34
Tensile strain at yield point		%	11
Flexural modulus		MPa	1400
Impact strength	without notch wg Charpy	kJ/m^2^	190
	notch wg Charpy		4

**Table 2:** Methodology of WPCs’ assessment.

Parameters	Unit	Value		
chink of screw-disk, W_s_	mm	0.3	1.7	3.0
rotational speed, n	rpm	12	26	40

**Table 3:** Results of the analysis of WPC composite images for different values of shear rate.

Parameters				Results			
				PP/Lignocel C120		PP/Lignocel 3-4	
γ ^·^ (s^−1^)	W_s_ (mm)	i (w_t_%)	n (rpm)	The Sum of All Areas of Agglomerates (mm^2^) ^·^	The Ratio of the Sum of the Agglomerate Areas to the Area Samples (%)	The Sum of All Areas of Agglomerates (mm^2^) ^·^	The Ratio of the Sum of the Agglomerate Areas to the Area Samples (%)
~800	0.3	35	26	2.599 × 10^3^	33	2.886 × 10^3^	55
~400	1.7	35	40	7.808 × 10^2^	10	3.860 × 10^3^	44
~70	3.0	35	26	1.540 × 10^3^	20	3.177 × 10^3^	45

**Table 4:** Results of the analysis of WPC composite images for different values of shear rate.

Parameters				Results			
				PP/Lignocel C 120		PP/Lignocel 3-4	
$\dot{\gamma }$ (S^−1^)	W_s_ (mm)	i (w_t_%)	n (rpm)	Size of the Area (mm^2^)	Number of Fields (pc)	Size of the Area (mm^2^)	Number of Fields (pc)
~800	0.3	35	26	≤40	1300	≤60	225
				40–75	8	60–135	2
				>75	7 and less	>135	
~400	1.7	35	40	≤2,5	1200		
				2,5–5	25		
				5–7,5	8		
				>10	4 and a less		
~70	3.0	35	26	≤7	520	≤70	289
				7–14	18	70–125	2
				14–22	8	>125	
				>22	4 and a less		

**Table 5:** Results of the analysis of WPC composite images for different values of rotational speed.

Parameters			Results			
			PP/Lignocel C 120		PP/Lignocel 3-4	
W_s_ (mm)	i (w_t_%)	n (rpm)	Size of the Area (mm^2^)	Number of Fields (pc)	Size of the Area (mm^2^)	Number of Fields (pc)
1.7	35	12	≤50	750	≤40	518
			50–120	5	75–150	2
			>120	4 and less	>190	>2
1.7	35	26	≤40	600	≤18	340
			40–96	4	18–35	8
			>96	3 and less	>50	3 and a less
1.7	35	40	≤10	150	≤90	335
			>24	1	>90	8 and less

## Data Availability

Data sharing not applicable.
